# A workflow-based, digitally gamified teaching model for pathology education: a pilot study

**DOI:** 10.1186/s12909-026-09786-4

**Published:** 2026-06-27

**Authors:** Ozge Ertener

**Affiliations:** Department of Pathology, Faculty of Medicine, İzmir University of Economics, İzmir, Türkiye

**Keywords:** Pathology education, Gamification, Medical education, Diagnostic reasoning, Workflow-based learning, Clinical transition, Undergraduate medical education

## Abstract

**Background:**

Pathology is a core component of undergraduate medical education, yet students often experience difficulty integrating preclinical knowledge into clinical practice. This challenge is particularly evident during the transition to clinical training, where understanding diagnostic workflows becomes essential. This study describes the development and implementation of a workflow-based, digitally gamified teaching model designed to support the integration of pathology knowledge and the development of diagnostic reasoning during the transition to clinical training.

**Methods:**

A ten-session teaching series was implemented for fourth-year medical students within a multidisciplinary clinical clerkship. The sessions were structured around the complete diagnostic pathway, from biopsy acquisition to pathology report interpretation. Digital and gamified learning activities were integrated throughout the series to support engagement and specific cognitive processes. Student perceptions were evaluated via a voluntary, anonymous postintervention survey, which included Likert-scale items and open-ended responses.

**Results:**

Sixteen students completed the survey (response rate 80%). The students reported high levels of engagement and positive learning experiences. All the respondents indicated an improved understanding of diagnostic workflows and pathology report interpretation. Gamified activities were perceived as helpful for maintaining engagement and supporting recall, particularly in relation to terminology and pattern recognition. Qualitative feedback highlighted high levels of engagement, memorability, and perceived educational value associated with the teaching approach.

**Conclusions:**

A workflow-based, digitally gamified teaching approach may provide a practical framework for integrating pathology knowledge and supporting diagnostic understanding during the transition to clinical training. The findings suggest that aligning teaching with authentic diagnostic workflows, combined with well-integrated interactive strategies, represents a potentially transferable approach for enhancing student engagement and supporting learning in this context.

**Supplementary Information:**

The online version contains supplementary material available at https://doi.org/10.1186/s12909-026-09786-4.

## Background

Pathology is a core component of undergraduate medical education, yet many students experience it as highly theoretical and difficult to connect with clinical practice. In many curricula, pathology content is delivered in system-based blocks across preclinical years, which often limits continuity and makes it difficult for students to integrate previously learned concepts during the transition to clinical training [1], a well-recognized challenge in medical education [2].

At the beginning of the fourth year, when students begin clinical clerkships, previously learned pathology concepts may be partially forgotten or difficult to apply within real diagnostic workflows and clinical decision-making contexts.

In our curriculum, pathology is delivered within a six-year undergraduate-entry medical curriculum leading to the Doctor of Medicine (MD) degree. During the first three years, students complete the preclinical phase, which is organized into three longitudinal course streams: Scientific Basis of Medicine (SBM), Clinical Basis of Medicine (CBM), and Human, Society and Planet (HSP). Within SBM, pathology-related concepts are first introduced during the second year through teaching on general mechanisms of disease, including cellular injury, inflammation, tissue repair, hemodynamic disorders, and neoplasia. During the third year, pathology teaching continues through integrated system-based modules that address organ-specific diseases within a multidisciplinary framework. This curriculum structure promotes horizontal integration across biomedical disciplines and vertical integration with clinically oriented learning activities delivered through CBM.

As a follow-up to this integrated preclinical teaching, the workflow-based teaching series introduced at the start of the fourth year was designed as an additional integrative educational component rather than a replacement for existing pathology teaching. Implemented immediately before students entered their clinical clerkships, the intervention aimed to reinforce previously learned knowledge, support knowledge consolidation during the transition to clinical training, and help students apply pathology concepts within authentic diagnostic workflows.

Educational approaches that promote active learning and learner engagement are commonly used to address challenges related to cognitive load and fragmented learning experiences [3]. A wide range of active methodologies and digitally supported instructional techniques have been increasingly incorporated into medical education to enhance learner participation and integration of knowledge [4]. Active learning strategies have been associated with improved learner engagement, knowledge retention, and development of clinical reasoning skills in medical education settings [5, 6].

Interactive digital tools and gamified instructional strategies are increasingly being incorporated into medical education to promote learner participation, provide immediate feedback, and support reinforcement of knowledge through active engagement [7, 8]. In pathology practice, digital workflow integration has become increasingly important for supporting coordinated diagnostic and laboratory processes [9]. Despite the increasing use of interactive and digital approaches, many teaching interventions still lack a clear structural framework that reflects how pathology is actually practiced in real clinical settings.

A workflow-based structure provides a practical approach to support knowledge integration by anchoring learning to the stepwise diagnostic pathway that a biopsy specimen follows, from acquisition and specimen handling to laboratory processing, microscopic evaluation, and pathology report interpretation. This pathway reflects established pathology workflow principles, in which diagnostic practice depends on coordinated pre-analytical, analytical, and reporting processes [9]. Whole-task instructional models propose that learning organized around authentic professional processes may support knowledge integration and transfer more effectively than fragmented content delivery, particularly in complex domains such as healthcare diagnostics [10]. Structuring learning around real diagnostic processes can help learners organize knowledge more effectively and better understand how individual laboratory steps contribute to diagnostic conclusions and clinical decision-making [11].

**Fig. 1:**
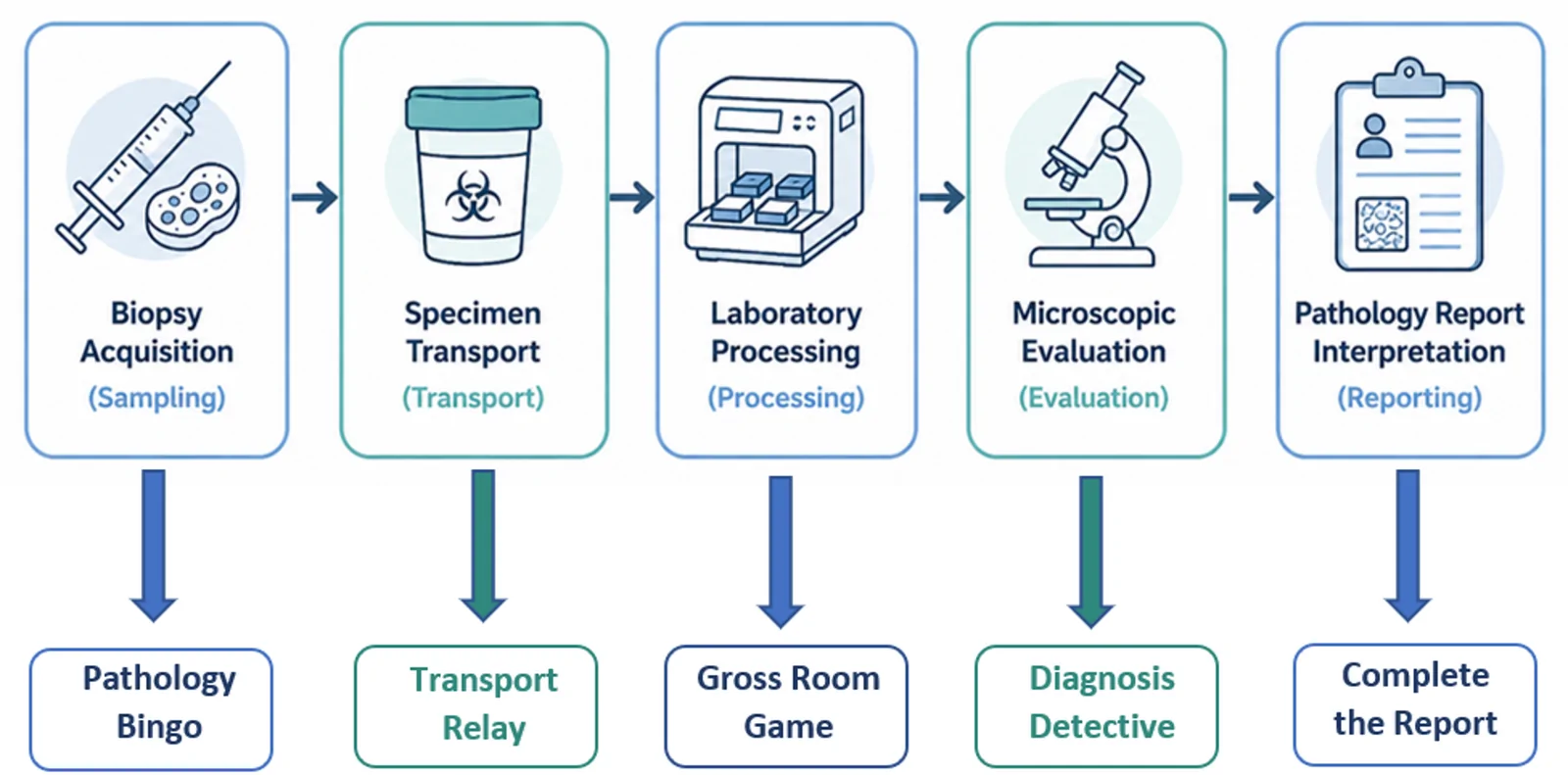
Workflow-based diagnostic pathway used to structure the teaching sessions The figure illustrates the stepwise diagnostic workflow of a biopsy specimen, from acquisition and specimen handling through laboratory processing, microscopic evaluation, and pathology report interpretation. Each stage represents a key component of the diagnostic process and provides a structural framework for supporting knowledge integration and clinical reasoning. The gamified learning activities incorporated within the teaching series are aligned with specific stages of the pathology workflow, including Pathology Bingo, Transport Relay, Gross Room Game, Diagnosis Detective, and Complete the Report. Mentimeter polling was implemented at the beginning of the lecture series to support cognitive readiness and prior knowledge activation and was therefore not linked to a specific workflow stage

On the basis of these educational principles, we developed and implemented a ten-session teaching series at the start of the fourth year. The series was structured around the complete biopsy-to-report diagnostic workflow (Fig. 1).

Digital gamification strategies were embedded throughout the series to support engagement and stepwise diagnostic reasoning, including anonymous real-time polling, terminology-based games, sequencing activities for specimen handling processes, pattern recognition tasks using digital microscopy images, and guided interpretation of real pathology reports (Table 1).

**Table 1:** Instructional design framework linking gamified teaching components to targeted diagnostic cognitive domains

Teaching Component	Educational Function	Target Cognitive Domain	Implementation Format
Mentimeter polling	Engagement and prior knowledge activation	Cognitive readiness/participation	Web-based; individual; smartphone/tablet
Pathology Bingo	Terminology reinforcement	Knowledge consolidation/recall	Paper-based; individual
Transport Relay	Workflow sequencing	Procedural diagnostic reasoning	Classroom activity; individual
Gross Room Game	Specimen orientation	Procedural diagnostic reasoning	Paper-based; individual
Diagnosis Detective	Visual pattern matching	Diagnostic pattern recognition	Small-group activity (5 groups of 4)
Complete the Report	Case-based report interpretation	Applied diagnostic reasoning	Individual; digital/paper

The table illustrates the relationships among instructional tools, their educational functions, and the targeted diagnostic cognitive domains addressed throughout the teaching series

This study describes the implementation of this workflow-based, digitally gamified teaching series and reports student perceptions of engagement, learning experience, and perceived understanding of pathology laboratory workflows and pathology report interpretation via a voluntary postintervention survey complemented by qualitative feedback.

This study also presents a structured and adaptable model that may be applied across pathology and other diagnostic disciplines.

## Methods

### Study design and setting

This study describes the implementation and evaluation of a pilot educational intervention in undergraduate pathology education. The intervention was conducted at İzmir University of Economics, Faculty of Medicine, within a fourth-year multidisciplinary clinical clerkship titled “Fundamentals of Clerkship” (CCS400), which integrates pathology teaching with other laboratory medicine disciplines, including microbiology and clinical biochemistry.

### Participants

The intervention was delivered to a cohort of fourth-year medical students (*n* = 20). Participation in teaching activities formed part of the standard curriculum. The evaluation was conducted via a voluntary, anonymous postintervention survey, which was completed by 16 students (response rate 80%).

### Educational intervention

The intervention consisted of a ten-session teaching series, with each session lasting 45 min, structured around the complete diagnostic workflow, following a biopsy specimen from acquisition to final pathology report interpretation (Fig. 1). The workflow sequence was informed by established pathology laboratory workflow frameworks [9].

The primary aim of the intervention was to support the integration of previously acquired pathology knowledge and to strengthen diagnostic reasoning during the transition to clinical training.

The teaching model combined workflow-based learning with digitally supported gamification strategies. Interactive tools and structured activities were embedded throughout the sessions to promote engagement, reinforce terminology, and support stepwise diagnostic reasoning.

Interactive components included anonymous real-time polling (Mentimeter) at the beginning of the lecture series to assess prior knowledge and support inclusive participation, Pathology Bingo designed to reinforce biopsy-related terminology, a transport relay game focused on specimen handling and pre-analytical workflow sequencing, a Gross Room game addressing specimen orientation and gross examination principles, a Diagnosis Detective activity linking real-life objects with microscopic structures, and a Complete the Report exercise involving pathology report interpretation. Gamified activities were implemented using a combination of web-based educational platforms, digital pathology resources, and paper-based exercises. Students participated synchronously during scheduled classroom sessions using their own smartphones or tablets when digital interaction was required. Most activities were completed individually, whereas the Diagnosis Detective activity was conducted collaboratively in five groups of four students. The Pathology Bingo activity was completed individually using paper-based materials.

Educational materials included illustrative biopsy images, pathology photographs, microscopic images, anonymized pathology report examples adapted for teaching purposes, and simulated diagnostic scenarios obtained from openly licensed educational resources and pathology education websites. No patient specimens were collected or analyzed as part of this educational intervention, and no identifiable patient information was used.

Each gamified component was intentionally aligned with specific diagnostic learning objectives, including cognitive readiness, terminology consolidation, workflow sequencing, diagnostic pattern recognition, and applied case-based reasoning (Table 1).

### Evaluation

Student perceptions of engagement, learning experience, and perceived diagnostic understanding were evaluated via an anonymous postintervention survey administered via Google Forms.

The survey was newly developed by the author for this study and was not derived from a previously published instrument. Survey items were designed to align with the educational objectives of the workflow-based teaching series and to evaluate student engagement, perceived learning, understanding of pathology workflows and pathology reports, perceived educational value of gamification, and overall usefulness of the intervention. The survey included Likert-scale items measuring agreement levels and an optional open-ended question for qualitative feedback. The English-language version of the survey is provided as Supplementary File 1.

### Data analysis

The survey data were analyzed using descriptive statistical methods. Quantitative responses were summarized via frequency distributions, and high-agreement responses were defined as Likert scores of 4 or 5. Open-ended responses were analyzed using descriptive content analysis. Responses were reviewed and grouped according to recurring ideas related to engagement, memorability, and perceived educational value, which were then summarized into descriptive categories.

## Results

### Participant response

The teaching series was delivered to a cohort of 20 fourth-year medical students. Sixteen students completed the voluntary postintervention survey, corresponding to a response rate of 80%. A descriptive summary of the student survey responses is presented in Table 2. The distribution of high-agreement responses (Likert scores of 4–5) across survey domains is summarized in Table 2.

**Table 2:** Student perceptions of engagement, learning experience, and perceived diagnostic understanding following the teaching series (*n* = 16)

Survey Domain	Survey Focus	Students reporting high agreement (Likert 4–5), *n* (%)
Active participation	Sessions encouraged active participation	15 (94%)
Digital engagement	Mentimeter supported participation	14 (88%)
Workflow understanding	Perceived understanding of laboratory workflow	16 (100%)
Report interpretation	Perceived confidence in interpreting pathology reports	16 (100%)
Clinical relevance	Perceived connection between pathology findings and clinical context	15 (94%)
Gamified learning experience	Gamified activities supported learning and retention	15 (94%)
Overall usefulness	Overall learning value of the teaching series	16 (100%)

Responses are based on a voluntary postintervention survey completed by 16 fourth-year medical students. The values represent the number and percentage of students selecting high-level agreement responses (Likert scores of 4 or 5) for each survey domain related to engagement, learning experience, and perceived diagnostic understanding

### Student engagement and perceived learning experience

The survey responses revealed high levels of student satisfaction and perceived engagement with the teaching approach. The majority of respondents selected high agreement responses (Likert scores of 4 or 5) for items related to active participation, perceived usefulness of the teaching sessions, and overall learning experience. High agreement levels were consistently observed across all survey domains, indicating a broadly positive perception of the teaching intervention.

The students reported that digital and gamified components supported sustained attention and engagement during the sessions. Anonymous real-time polling using Mentimeter was consistently rated as helpful for participation, particularly by students who may be less likely to contribute verbally in traditional classroom settings. A single low-agreement response (Likert score of 1) was observed for the Mentimeter engagement item, whereas no low-agreement responses were recorded across the remaining survey domains.

### Perceived understanding of diagnostic workflow and reporting

The students reported strong improvements in their understanding of pathology laboratory workflows and the structure of pathology reports. Most respondents selected high agreement ratings for items related to understanding specimen handling processes, laboratory workflow sequencing, and interpretation of pathology report components.

Students also reported increased confidence in approaching pathology reports and understanding the relationship between laboratory findings and clinical decision-making contexts.

### Evaluation of gamified teaching components

Gamified learning activities were positively evaluated across all the components. Pathology Bingo and Diagnosis Detective were frequently described in open-ended responses as enjoyable and supportive for remembering key concepts. Sequencing-based activities, including the transport relay game, were reported to support the understanding of stepwise laboratory processes.

### Qualitative feedback

Descriptive content analysis of the open-ended responses identified three recurring categories: engagement, memorability, and perceived educational value.

Engagement referred to students’ increased interest and participation during the sessions, particularly in relation to the interactive and gamified activities. One student commented that “the games increased all of our interest and engagement in the course.”

Memorability reflected students’ perceptions that the learning experience was enjoyable and likely to be retained over time. As one participant stated, the sessions were “very enjoyable and memorable.”

Perceived educational value encompassed students’ views regarding the usefulness and effectiveness of the teaching approach. Several comments described the sessions as informative, useful, and productive, with one student noting that “the course was truly enjoyable and informative.”

Overall, the qualitative feedback was consistent with the quantitative findings and suggested that students perceived the workflow-based, gamified teaching approach positively.

### Student interest in pathology laboratory environments

In addition to the structured survey responses, informal face-to-face feedback collected during and after the teaching series indicated increased interest in pathology laboratory practice. Several students expressed interest in observing laboratory workflows directly and suggested that a laboratory visit could further support the understanding of diagnostic processes. For example, one student commented that while the sessions were clear and enriched by interactive methods, observing the pathology laboratory in practice could help them better understand workflow processes and support career-related decision-making. In response to this interest, a voluntary laboratory observation session has been planned for students who wish to participate. 

The detailed quantitative results for each survey domain are presented in Table 2.

## Discussion

This study describes the development and initial implementation of a workflow-based, digitally gamified teaching model within a fourth-year clinical clerkship designed to support pathology learning during the transition from preclinical to clinical education. The findings indicate high levels of student engagement and positive perceptions of learning, particularly in relation to understanding diagnostic workflows and interpreting pathology reports. These results suggest that structuring pathology education around authentic diagnostic processes, supported by well-integrated interactive strategies, may provide an effective approach to addressing fragmentation in undergraduate medical education.

One of the key contributions of this study is the introduction of a workflow-based instructional framework that aligns pathology teaching with real diagnostic processes, enabling students to integrate previously learned knowledge within a coherent and clinically meaningful structure. While previous studies in medical education have explored active learning and gamification [7] and digital approaches to pathology education [12], fewer have integrated these approaches within a structured representation of the complete diagnostic pathway. By organizing learning along the biopsy-to-report continuum, the present model provides a coherent structure through which students can integrate previously acquired knowledge and develop a more comprehensive understanding of how individual laboratory steps contribute to clinical decision-making [13].

The observed improvements in students’ perceived understanding of diagnostic workflows are consistent with whole-task instructional design principles [10], which emphasize that learning structured around complete professional tasks can facilitate knowledge integration and transfer. In this context, the workflow-based model appears to support the integration of previously fragmented preclinical knowledge into a more clinically meaningful framework [14], enabling learners to better understand both the sequence and the interdependence of diagnostic processes.

In this study, gamification functioned as a supporting rather than central pedagogical strategy. Gamified activities were well received by students and appear to support engagement and recall [7, 15, 16]. However, their effectiveness appears to depend largely on their integration within a coherent instructional structure. Rather than functioning as isolated engagement tools, these activities were aligned with specific stages of the diagnostic process and targeted cognitive domains such as knowledge consolidation, procedural reasoning, and pattern recognition and the application of this knowledge in clinical contexts.

Importantly, the intervention was implemented at a critical transition point in medical education, when students moved from preclinical knowledge acquisition to clinical application. This transition is often associated with difficulties in applying previously learned concepts within real clinical contexts [1]. The findings suggest that structured, workflow-based teaching interventions may support this transition by reinforcing prior knowledge and situating it within an authentic and contextually meaningful diagnostic framework [17]. In doing so, the model contributes not only to knowledge integration but also to the development of diagnostic reasoning [18, 19]. Furthermore, the model may be adaptable to other laboratory-based and diagnostic disciplines in which workflow understanding and knowledge integration are essential.

This study has several limitations that should be considered. The sample size was limited to a single cohort of students within one institution, and the findings are based on self-reported perceptions. In addition, the absence of objective performance measures limits the ability to draw conclusions about actual learning outcomes. The study also did not include a comparison or control group, which may have strengthened conclusions regarding the educational effectiveness of the intervention. Furthermore, the intervention was implemented within the context of Anatomical Pathology only. Although the workflow-based framework may be applicable to other laboratory-based and diagnostic disciplines, including Clinical Biochemistry, Microbiology, and related laboratory medicine specialties, this was not evaluated in the present study. As such, the findings should be interpreted as preliminary and exploratory. Future studies incorporating objective assessment methods and larger, multi-institutional cohorts would strengthen the evidence base for this approach.

Despite these limitations, the study has notable strengths, including a clearly defined instructional model, a detailed description of implementation, and the integration of educational theory with practical application. The structured alignment between workflow-based learning, cognitive processes, and gamified activities represents a meaningful contribution to the field of pathology education.

Future research should explore the impact of this model on objective learning outcomes, long-term knowledge retention, and clinical performance. In addition, the adaptability of the workflow-based framework to other diagnostic disciplines and its potential integration with emerging technologies, such as artificial intelligence-supported learning environments, warrant further investigation.

## Conclusions

This study describes the development and initial implementation of a workflow-based, digitally gamified teaching model designed to support pathology learning during the transition from preclinical to clinical education. The findings suggest that structuring teaching around authentic diagnostic workflows, combined with well-integrated interactive strategies, can enhance student engagement and support the integration of previously acquired knowledge without replacing existing preclinical instruction.

When implemented within a multidisciplinary clerkship context, the model also supports both vertical and horizontal integration across laboratory medicine disciplines, providing a more coherent and clinically meaningful learning experience. By addressing a critical gap at the transition to clinical training, this approach offers a practical and adaptable framework that may be transferable to other diagnostic and laboratory-based educational settings.

Further research is needed to evaluate the impact of this model on objective learning outcomes, long-term knowledge retention, and its applicability across different educational contexts.

## Supplementary Information

Supplementary Material 1.

## Data Availability

The datasets generated and/or analyzed during the current study are available from the corresponding author upon reasonable request.

## Abbreviations

SBM: Scientific Basis of Medicine
CBM: Clinical Basis of Medicine
CCS400: Fundamentals of Clerkship
PBL: Problem-based learning

## Declarations

Participation in the survey was voluntary and anonymous. Informed consent to participate was obtained from all participants prior to completion of the survey.

## References

[1] Prince KJAH, van de Wiel MWJ, Scherpbier AJJA, van der Vleuten CPM, Boshuizen HPA. A qualitative analysis of the transition from theory to practice in undergraduate training in a PBL-medical school. Adv Health Sci Educ Theory Pract. 2000;5(2):105–16. 10.1023/A:1009873003677. 12386467

[2] Brauer DG, Ferguson KJ. The integrated curriculum in medical education: AMEE Guide 96. Med Teach. 2015;37(4):312–22. 10.3109/0142159X.2014.970998. 25319403

[3] Sweller J, van Merriënboer JJG, Paas F. Cognitive architecture and instructional design: 20 years later. Educ Psychol Rev. 2019;31(2):261–92. 10.1007/s10648-019-09465-5.

[4] Peña S, Martinez-Santander C, Cantos-Reyes V, Frausto Rojas M. Active methodologies and didactic techniques in medical education: a systematic review. Educ Médica. 2025;26:101040. 10.1016/j.edumed.2025.101040.

[5] Parsad V, Divakaran J. Active learning in medical education: a brief overview of its benefits. MAR Pathol Clin Res. 2025;2:4. 10.5281/zenodo.14709388.

[6] Freeman S, Eddy SL, McDonough M, Smith MK, Okoroafor N, Jordt H, Wenderoth MP. Active learning increases student performance in science, engineering, and mathematics. Proc Natl Acad Sci U S A. 2014;111(23):8410–5. 10.1073/pnas.1319030111. 24821756 PMC4060654

[7] van Gaalen AEJ, Brouwer J, Schönrock-Adema J, Bouwkamp-Timmer T, Jaarsma ADC, Georgiadis JR. Gamification of health professions education: a systematic review. Adv Health Sci Educ Theory Pract. 2021;26(2):683–711. 10.1007/s10459-020-10000-3. 33128662 PMC8041684

[8] Ellaway RH, Masters K. AMEE Guide 32: e-Learning in medical education. Med Teach. 2008;30(5):455–73. 10.1080/01421590802108331. 18576185

[9] Fraggetta F, L’Imperio V, Ameisen D, Carvalho R, Leh S, Kiehl TR, et al. Best practice recommendations for the implementation of a digital pathology workflow in the anatomic pathology laboratory by the European Society of Digital and Integrative Pathology (ESDIP). Diagnostics (Basel). 2021;11(11):2167. 10.3390/diagnostics11112167. 34829514 PMC8623219

[10] van Merriënboer JJG, Kirschner PA. Ten Steps to Complex Learning: A Systematic Approach to Four-Component Instructional Design. 3rd ed. New York (NY): Routledge; 2018.

[11] Herrington J, Reeves TC, Oliver R. Authentic learning environments. In: Spector JM, Merrill MD, Elen J, Bishop MJ, editors. Handbook of Research on Educational Communications and Technology. New York (NY): Springer; 2014. pp. 401–12. 10.1007/978-1-4614-3185-5_32.

[12] Hassell LA, et al. Pathology education powered by virtual and digital transformation: now and the future. Arch Pathol Lab Med. 2023;147(4):474–91. 10.5858/arpa.2021-0473-RA. 35878400

[13] Xie H, et al. Application of a laboratory-result-oriented modular teaching model in the training of clinical hematology laboratory interns. Front Med (Lausanne). 2025;12:1612934. 10.3389/fmed.2025.1612934. 40901509 PMC12399513

[14] Dolmans DHJM, De Grave W, Wolfhagen IHAP, van der Vleuten CPM. Problem-based learning: future challenges for educational practice and research. Med Educ. 2005;39(7):732–41. 10.1111/j.1365-2929.2005.02205.x. 15960794

[15] Dichev C, Dicheva D. Gamifying education: what is known, what is believed and what remains uncertain. Int J Educ Technol High Educ. 2017;14:9. 10.1186/s41239-017-0042-5.

[16] Chi MTH, Wylie R. The ICAP framework: linking cognitive engagement to active learning outcomes. Educ Psychol. 2014;49(4):219–43. 10.1080/00461520.2014.965823.

[17] Koens F, Mann KV, Custers EJFM, Ten Cate OTJ. Analyzing the concept of context in medical education. Med Educ. 2005;39(12):1243–9. 10.1111/j.1365-2929.2005.02338.x. 16313584

[18] Norman G. Research in clinical reasoning: past history and current trends. Med Educ. 2005;39(4):418–27. 10.1111/j.1365-2929.2005.02127.x. 15813765

[19] Eva KW. What every teacher needs to know about clinical reasoning. Med Educ. 2005;39(1):98–106. 10.1111/j.1365-2929.2004.01972.x. 15612906

