# Supplementary material for: A workflow-based, digitally gamified teaching model for pathology education: a pilot study
Source: BMC Med Educ. 2026 Jun 27;26:1477. doi: 10.1186/s12909-026-09786-4 (PMC13584541; doi:10.1186/s12909-026-09786-4)
Supplement: Supplementary file 1 [file 12909_2026_9786_MOESM1_ESM.docx]

**Supplementary File 1. Post-intervention survey questionnaire**

The survey was originally administered in a bilingual format (Turkish/English) to facilitate clarity and student comprehension. The English-language version is provided here for supplementary documentation purposes.

The following introductory statement was presented at the beginning of the survey:

“This survey was designed to collect student feedback regarding the workflow-based, digitally gamified pathology teaching series implemented within the CCS400 clerkship. Responses were collected anonymously and participation was voluntary.”

**Response Scale:**

1 = Strongly disagree

2 = Disagree

3 = Neutral

4 = Agree

5 = Strongly agree

**Survey Items**

1. I felt actively involved during the teaching sessions.

2. The use of Mentimeter at the beginning of the sessions increased my attention and engagement.

3. I gained a better understanding of pathology laboratory workflows.

4. I improved my understanding of pathology report structure and interpretation.

5. The teaching sessions helped me relate pathology knowledge to clinical applications.

6. The Pathology Bingo activity made learning more enjoyable.

7. Gamified activities helped me retain information more effectively.

8. The overall structure and teaching methods met my expectations.

9. Overall, I found the teaching series useful.

**Open-ended Question**

If you have any additional comments or suggestions about the course, please share them below. (Optional)
